# Effects of physical activity on externalizing problems in adolescents with attention deficit hyperactivity disorder: the chain mediating effects of school connectedness and self-control

**DOI:** 10.3389/fpsyg.2026.1850288

**Published:** 2026-06-25

**Authors:** Tiantian Yue, Ke Zhou, Jiabing Zhou, Cheng Zheng

**Affiliations:** 1 School of Physical Education, Henan University, Kaifeng, Henan, China; 2 School of Physical Education, Guangdong University of Petrochemical Technology, Maoming, Guangdong, China; 3 School of Sports Science and Physical Education, Nanjing Normal University, Nanjing, Jiangsu, China

**Keywords:** attention deficit hyperactivity disorder, externalizing problems, physical activity, school connectedness, self-control

## Abstract

**Objective:**

The prevalence of externalizing problems in adolescents with Attention Deficit Hyperactivity Disorder (ADHD) has shown a steady upward trend in recent years. Physical activity (PA), recognized as a key strategy for enhancing emotional regulation and self-control, may be negatively associated with these behaviors. This current study aims to investigate the mechanisms through which Physical activity is associated with externalizing problems in ADHD adolescents, with a particular focus on the chain mediating effects of school connectedness and self-control within this relationship.

**Methods:**

A total of 517 adolescents with ADHD were recruited using a combination of purposive and convenience sampling. Participants were assessed using the Physical Activity Rating Scale, Strengths and Difficulties Questionnaire, School Connectedness Scale, and Self-Control Scale. Data were analyzed using SPSS 27.0 and Process 4.1 software to examine the internal relationships among the four variables.

**Results:**

(1) Physical activity was negatively associated with externalizing problems and positively associated with school connectedness and self-control. (2) Both school connectedness and self-control independently mediated the association between physical activity and externalizing problems in adolescents with ADHD. (3) Additionally, a sequential (chain) mediation effect was identified, whereby physical activity was related to externalizing problems through both mediators in sequence.

**Conclusion:**

This current study highlights the significant potential of physical activity to be negatively associated with externalizing problems in adolescents with ADHD and its underlying mediating mechanisms. The findings suggest that promoting appropriate PA and enhancing school connectedness and self-control may be promising strategies associated with improved mental health outcomes in this population. Future research should consider age-related characteristics and individual preferences for types of physical activity.

## 1 Introduction

Adolescent mental health has become a central focus in global public health (Golden et al., 2024). Among neurodevelopmental disorders, Attention Deficit Hyperactivity Disorder (ADHD) stands out as a major challenge in both clinical interventions and educational practice (Guha, 2014). Epidemiological studies suggest that the global prevalence of ADHD is approximately 5.26% (Posner et al., 2020), with the rate among Chinese adolescents estimated at 6.4% (Li et al., 2022), indicating a rising trend in recent years. According to the (Guha, 2014), ADHD is characterized by symptoms of inattention, hyperactivity, and impulsivity, often accompanied by impairments in self-regulation, social interactions, and oppositional behaviors (Posner et al., 2020). These symptoms typically emerge in childhood and may be persist into adolescence and adulthood (Faraone et al., 2006). Notably, adolescence is a high-risk period for ADHD comorbidities, including anxiety disorders, conduct disorders, and other psychiatric conditions (Agnew-Blais et al., 2016). Studies indicate that about 5% of adolescents with ADHD also exhibit externalizing problems (Luo et al., 2020), which maybe have long-lasting effects on individual development and social adaptation, making it a critical area for further research.

Externalizing problems refer to overt, maladaptive behaviors such as hyperactivity, impulsivity, aggression, and rule-breaking, which arise from difficulties in self-regulation (Goodman et al., 2010). These behaviors are closely linked to the clinical symptoms of ADHD and often result in negative consequences, including violations of social norms, property damage, and aggression toward others. Compared to typically developing children, adolescents with ADHD are significantly more likely to display externalizing problems, which hinder their school adaptation, leading to academic disengagement, difficulty in peer relationships, and even involvement in deviant behaviors like bullying (Chandler et al., 2016; Jia et al., 2024). Given the severity of these issues, identifying risk and protective factors is essential for addressing externalizing problems and promoting healthier development in ADHD adolescents.

Physical activity (PA) has emerged as a cost-effective, easily implemented strategy associated with fewer externalizing behaviors in adolescents (Wu et al., 2025). Previous research suggests that PA is positively linked to executive functions, such as inhibitory control and working memory, by regulating the neuroendocrine system and promoting the development of the prefrontal cortex in individuals with ADHD (Christiansen et al., 2019). High-intensity aerobic exercise, in particular, has been linked to reductions in impulsivity and aggressive behaviors. Furthermore, studies indicate a positive correlation between PA levels and self-control across various populations (De Greeff et al., 2018). For example, college students engaging in high-intensity PA demonstrate significantly better self-control than those participating in low-intensity activities (Ye et al., 2025). As a structured, rule-based activity, PA is associated with self-control, reduced aggression, and fewer externalizing behaviors, particularly in ADHD adolescents who struggle with executive function deficits (Sun et al., 2024). Additionally, school-based PA, such as participation in team sports and recess activities, fosters greater peer and teacher interactions and strengthens school connectedness. Strong school connectedness has been shown to improve psychological wellbeing, increase feelings of security, and reduce negative emotions associated with externalizing problems (Liu et al., 2024).

Collectively, existing research has documented associations between physical activity (PA) and fewer externalizing problems, as well as improved psychosocial outcomes, primarily among typically developing adolescents. However, most of these findings cannot be directly generalized to adolescents with ADHD, leaving the relationship between PA and externalizing problems in this clinical population unclear. To date, few studies to date have examined the serial mediation of school connectedness and self-control in this association. Therefore, the aim of the current study is to explore the associations between PA, externalizing problems, school connectedness, and self-control among adolescents with ADHD, specifically examining the serial mediating roles of school connectedness and self-control.

### 1.1 Physical activity and externalizing problems

PA refers to structured, purposeful, and repetitive physical exercise aimed at maintaining physical health, has been documented as a protective factor for the psychological wellbeing of adolescents (Caspersen et al., 1985). Existing studies have demonstrated that consistent PA not only alleviates symptoms of anxiety and depression but also significantly reduces the likelihood of externalizing behaviors, such as aggression and impulsivity (Hunter-Rue et al., 2024). This dual regulatory effect reflects the multifaceted mechanisms through which PA impacts individual functioning (Liang et al., 2025; Sahithya and Kashyap, 2023). From a theoretical standpoint, The Catharsis Theory posits that PA offers adolescents a means of expressing and releasing negative emotions (Scheff, 2012). PA has been shown to influence neurotransmitter regulation, with substantial evidence indicating that engagement in PA is associated with increased dopamine levels (Marques et al., 2021). These changes not only alleviate negative emotions like depression and anxiety but also improve cognitive function and self-regulation, thereby reducing the likelihood of impulsive and aggressive behaviors (Martinez and Martins, 2024). Empirical evidence supports these findings, with two studies on university students demonstrating a significant negative correlation between PA and aggressive behavior (Yu et al., 2024). Systematic reviews and meta-analyses further confirm that PA interventions are effective in reducing aggression in children and adolescents, indicating that PA is more effective in alleviating externalizing symptoms in adolescents with ADHD (Ouyang and Liu, 2023).

Furthermore, from a psychological perspective, PA is a collective, rule-based activity that fosters behavioral regulation through the development of sportsmanship (Hagger and Chatzisarantis, 2008). The emphasis on rule adherence and teamwork within PA strengthens individual organizational skills and self-discipline, with this regulatory influence gradually internalizing into a stable sense of self-management and self-control (Mandolesi et al., 2018). This awareness targets the externalizing behaviors in adolescents with ADHD caused by deficits in self-control, effectively inhibiting the occurrence of these behaviors. In related research focusing on this group, a 12-week combined intervention of aerobic exercise and cognitive engagement demonstrated that PA significantly improved inhibitory control in children with ADHD, with this effect persisting for at least 12 weeks (Liang et al., 2022). Nevertheless, existing research remains contentious. While some studies suggest that PA is associated with improved externalizing behaviors in adolescents with ADHD, a meta-analysis has confirmed the emotional benefits of PA but found no sustained association with externalizing behaviors during long-term follow-up (Recchia et al., 2023). In conclusion, previous research has largely overlooked the relationship between PA and externalizing behaviors in adolescents with ADHD and has not systematically examined the psychological mediators involved. However, the current study aims to further explore the relationship between PA and externalizing behaviors in adolescents with ADHD, building upon prior research.

### 1.2 Mediating role of school connectedness

School connectedness refers to the degree of respect, acceptance, and support an individual perceives within the school environment, reflecting the emotional bonds between the individual, teachers, and peers (Centers for Disease Control and Prevention, 2009). According to Social Support Theory, the various forms of support derived from social relationships are vital resources for adapting to one's environment, particularly in the context of psychological and social adjustment (Cassel, 1976). For adolescents with ADHD, school serves as a central domain for their daily life, learning, and social interactions. School connectedness represents a form of social support within this context (Coyne-Foresi, 2015). PA conducted in this environment not only provides enjoyable experiences for adolescents with ADHD but also fosters the development of empathy and social cognition through interactive mechanisms such as role division and goal-sharing. Additionally, the non-verbal communication and emotional synchronization phenomena that occur during PA may be activate the mirror neuron system, promoting group cohesion. This interactive environment enhances relationships among students, fostering a stronger sense of belonging and ultimately improving school connectedness. These associations have been empirically supported by existing research (Ferrari et al., 2021).

From the perspective of the buffering effect in Social Support Theory, school connectedness, as a stable campus support resource, has been associated with reduced externalizing behaviors in adolescents with ADHD. Specifically, among the dimensions of teacher support, peer support, and school belonging, school belonging, as a deeper form of emotional support, has the most significant negative predictive effect on adolescents' externalizing problems (Bond et al., 2007). It is noteworthy that peer acceptance and teacher guidance do not directly influence externalizing behaviors. Instead, they act as supportive pathways to school belonging, reducing feelings of alienation. Social Support Theory highlights the synergistic effects of multi-source support, with research indicating that positive school connectedness, alongside family and community support, is associated with fewer externalizing behaviors (Santisteban et al., 2012). However, research on the relationship between school connectedness and externalizing behaviors in adolescents with ADHD is relatively limited. However, The current study hypothesizes that school connectedness mediates the relationship between PA and externalizing behaviors in adolescents with ADHD.

### 1.3 Mediating role of self-control

Self-control refers to an individual's ability to override immediate impulses, habits, or automatic responses, consciously regulating their behavior to align with social norms and long-term goals (De Ridder et al., 2012). ADHD, a neurodevelopmental disorder that typically manifests in childhood, is characterized by a core deficit in self-control, which may serve as a key contributor to the development of externalizing problems (Wang et al., 2017). The strength model of self-control suggests that an individual's self-control relies on limited psychological resources (Li et al., 2015). Frequent exertion of self-control is associated with the depletion of these resources, causing psychological resource exhaustion. The model compares psychological resources to muscles, proposing that they are depleted with sustained self-control but may be regenerated and strengthened through active training. This hypothesis supports the idea that PA enhances the recovery of psychological resources, reduces excessive depletion, and thus facilitates the expansion and replenishment of self-control resources. Cognitive neuroscience research further supports this view, indicating that low-intensity PA may be induce cortical activation in the dorsolateral prefrontal cortex, left prefrontal cortex, and prefrontal polar regions. The functional activity in these areas is strongly linked to enhanced self-control, indicating a positive relationship between PA and self-control ability (Boat and Cooper, 2019).

The dual-system model of self-control further refines the underlying mechanism through which self-control relates to externalizing problems (Steinberg, 2010). This model divides self-control into two systems; the impulsive system and the control system. The control system is associated with the effortful process of achieving good self-control, linked to high self-control and reduced impulsive decision-making (Dvorak et al., 2011). Higher self-control has been associated with fewer externalizing problems in adolescents. In contrast, the impulsive system corresponds to the impulsive process of poor self-control, associated with low self-control and potential impulsive behaviors, which correlate with externalizing problems in adolescents (McDermott et al., 2017). Thus, good self-control serves as a protective factor in reducing externalizing problems in adolescents. However, considering the core deficits in adolescents with ADHD and the effects of PA on improving self-control, it is plausible that PA could improve self-control in this population, which may, in turn, be associated with reduced externalizing behaviors.

### 1.4 Chain mediation of school connectedness and self-control

From the perspective of person-environment interaction, the school connectedness effect does not exist in isolation but is moderated by individual characteristics such as self-control (Fergus and Zimmerman, 2005). There are two hypotheses regarding the interaction between school connectedness and self-control: one suggests that the positive correlates of school connectedness is more pronounced in adolescents with high self-control, while the other proposes that the improvement effect is more significant in those with low self-control (Wang, 2009). For adolescents with ADHD, their insufficient self-control makes them a typical group for the protective weakening model. In this model, PA amplifies the intrinsic connection between school connectedness and self-control through a progressive pathway. First, PA creates a structured peer interaction environment for adolescents with ADHD, enhancing school connectedness through teamwork (McNamara et al., 2015). Building on this, the increased school connectedness strengthens individuals' identification with and adherence to group rules, thereby enhancing self-control. Ultimately, both factors work synergistically to correlate with fewer externalizing problems. Therefore, based on empirical research on the protective weakening model, it may be preliminarily inferred that the chain transmission between school connectedness and self-control may be the key pathway through which PA is associated with externalizing problems in adolescents with ADHD. However, clarifying the relational logic between school connectedness, self-control, and externalizing problems in adolescents with ADHD provides important theoretical support for this current study.

### 1.5 Hypotheses of this study

**Figure 1:**
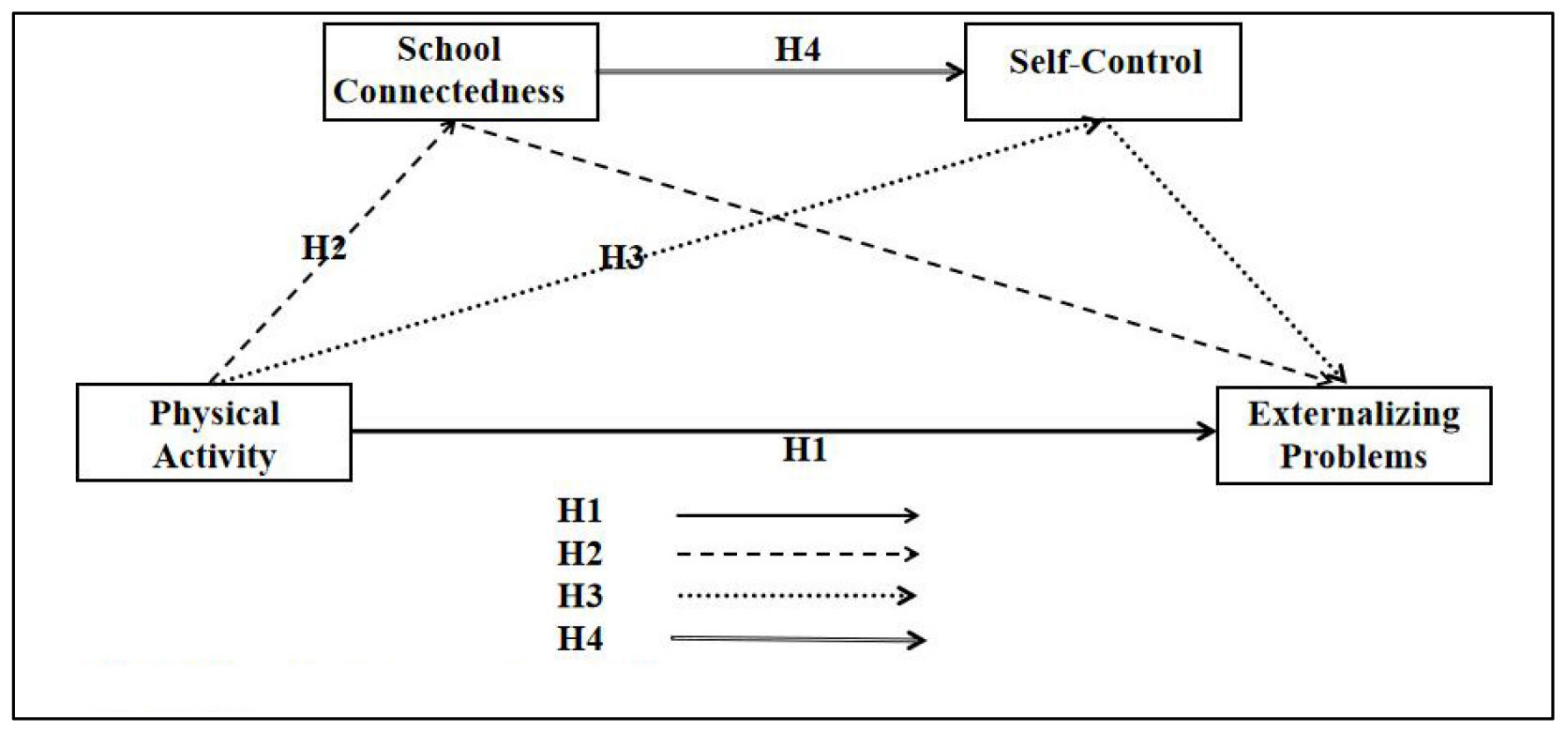
The theoretical model diagram.

This current study aims to explore the chain-mediated effects of school connectedness and self-control in the relationship between PA and externalizing problems in adolescents with ADHD, with the goal of deepening the understanding of how PA relates to externalizing problems through psychosocial mechanisms in this population. While existing research has indicate that PA, school connectedness, and self-control are closely related to externalizing problems, the specific mechanisms of these factors in adolescents with ADHD remain unclear. Therefore, this current study is expected to provide theoretical support and practical insights for the prevention and intervention of externalizing problems in adolescents with ADHD. Based on the existing literature, the following research hypotheses are proposed (see Figure 1):

H1: PA is negatively associated with externalizing problems in adolescents with ADHD.
H2: School connectedness plays an independent mediating role between PA and externalizing problems in adolescents with ADHD.
H3: Self-control plays an independent mediating role between PA and externalizing problems in adolescents with ADHD.
H4: School connectedness and self-control play a chain mediating role between PA and externalizing problems in adolescents with ADHD.

## 2 Materials and methods

### 2.1 Sample

This current study used the sample size formula: N $=4{\left(\frac{\mu_{\alpha }S}{\delta }\right)}^{2}$ where μα is the μ value corresponding to the standard level α. In the current study, α = 0.001, and μα = 3.29. The permissible error was set between [0.25 s, 0.50 s], resulting in a sample size range of 173–692 participants. For mediation analysis, a sample size exceeding 200 participants is required. Therefore, this current study selected 517 adolescents diagnosed with ADHD from local integrated schools, special education schools, and rehabilitation centers as research participants. Inclusion criteria were as follows: (1) Children and adolescents aged 6–16 with an ADHD diagnosis made by pediatric psychologists and experienced developmental-behavioral pediatricians at local hospitals based on the criteria in the fifth edition of the Diagnostic and Statistical Manual of Mental Disorders (DSM-5) (Bandyopadhyay Prasanta et al., 2014); (2) IQ ≥ 85 based on the Wechsler Intelligence Scale for Children (WISC-IV) and no current medication treatment, to eliminate potential confounding effects of psychotropic medications (e.g., stimulants, antidepressants) on psychological and behavioral outcomes, which may alter neurotransmitter levels, attention, and impulsivity and compromise the validity of results; (3) Right-handed with normal uncorrected or corrected vision, no color blindness, voluntarily participating and able to adhere to the study, with informed consent obtained from the child's guardian, to reduce variability in neuropsychological and behavioral measures related to hemispheric lateralization and ensure consistency and comparability of responses across motor and cognitive tasks.

**Figure 2:**
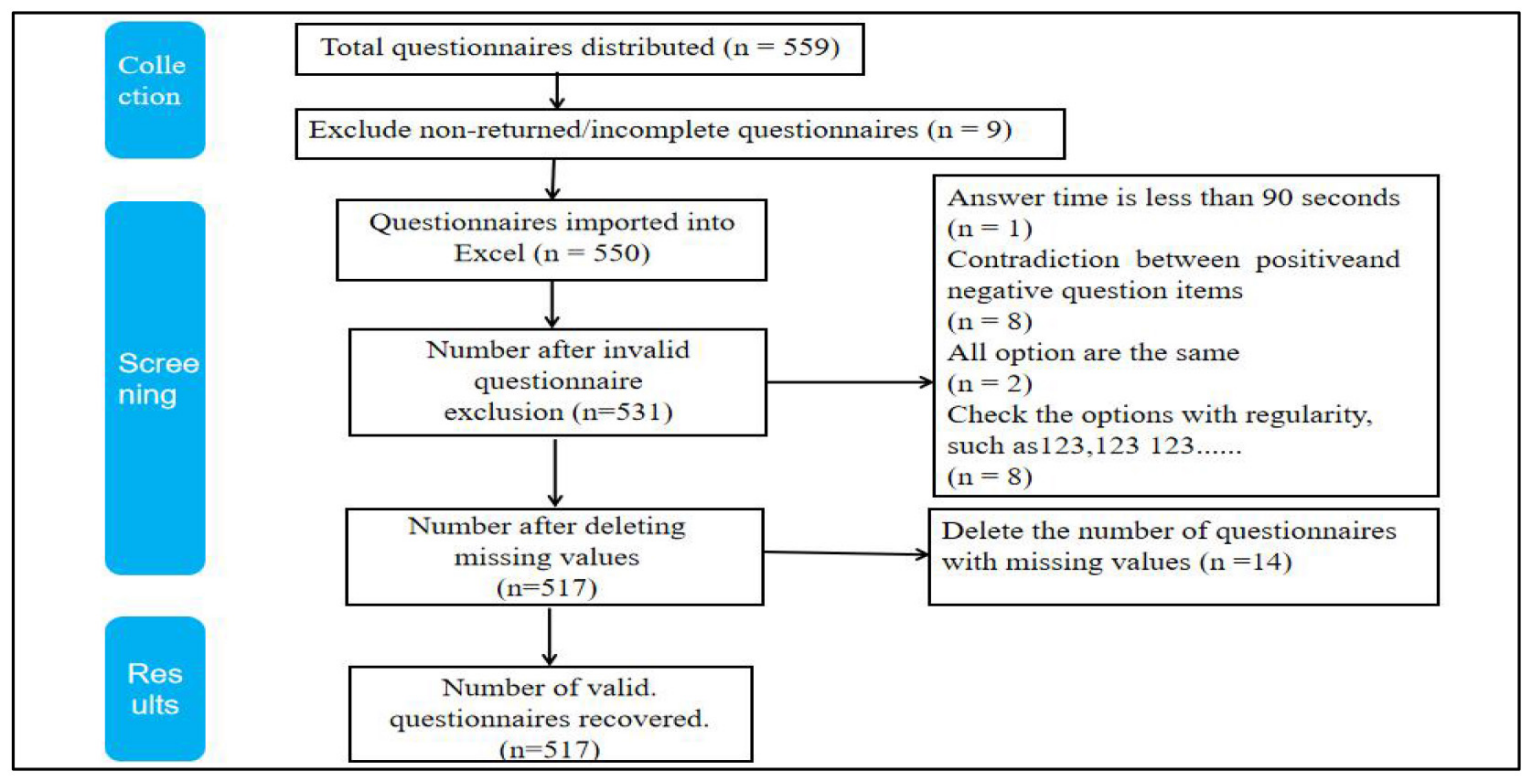
Steps in the screening process for research samples.

Exclusion criteria: (1) Full-scaleIQ < 85 on the Wechsler Intelligence Scale; (2) Diagnosis of any neurodevelopmental disorders other than ADHD (e.g., autism spectrum disorder, intellectual disability); (3) Diagnosis of major physical or neurological diseases; (4) Diagnosis of severe mental disorders, such as schizophrenia. Exclusion and dropout criteria: (1) Inability to participate in the survey due to other reasons, including but not limited to contact issues (inaccurate information, inability to reach), refusal to participate, and skepticism about the current study; (2) Withdrawal from the study or loss to follow-up. A total of 559 questionnaires were distributed, and 517 valid questionnaires were returned, resulting in a response rate of 92.5%. The specific screening process is shown in Figure 2. Among the 517 valid questionnaires returned, 282 (54.5%) were male and 235 (45.5%) were female. The average age of the adolescents was 12.73 ± 1.14 years, with detailed demographic characteristics presented in Table 1.

**Table 1:** Distribution of basic information on adolescents (*N* = 517).

Demographic variables		Number	Proportion %
Age	12.73 ± 1.14	517	100%
Gender	Male	282	54.5%
	Female	235	45.5%
Grade leve	Sixth grade	77	14.9%
	First year	153	29.6%
	Second year	166	32.1%
	Third year	121	23.4%
Place of birth	Rural	333	64.4%
	Urban	184	35.6%
Only child or not	Yes	180	34.8%
	No	337	65.2%
Boarding or not	Yes	305	59.0%
	No	212	41.0%
Behavioral intervention or not	Yes	367	71.0%
	No	150	29.0%
Year of diagnosis	0–3 years	420	81.2%
	More than 3 years	97	18.8%

### 2.2 Procedure

This current study was approved by the Biomedical Research Ethics Committee of Henan University. Participants were selected using purposive and convenience sampling. Written informed consent was obtained from participants or their legal guardians if minors. Qualified researchers and pre-screened assistants conducted enrollment and core indicator assessments. Prior to completing self-report scales and structured questionnaires, participants were fully briefed on the current study's purpose and requirements. All personnel underwent specialized training and consistency tests to ensure standardized procedures and data accuracy. After completion, data integrity was verified before being stored in a centralized study database.

### 2.3 Measurement

#### 2.3.1 Physical activity

The current study used the Physical Activity Rating Scale (PARS-3), revised by (Liang 1994), which was originally developed by Japanese psychologist Koki Hashimoto, to assess the PA levels of adolescents with ADHD. This scale has been widely applied in related research and evaluates three dimensions of PA (Wu et al., 2024); exercise intensity, duration, and frequency. Each item is rated on a five-point Likert scale, with intensity and frequency scored from 1 to 5, while duration is rated from 0 to 4. The total PA score is calculated using the formula: PA amount = Intensity × Duration × Frequency. Based on the following standards ≤ 19 for low activity, 20–42 for moderate activity, and ≥43 for high activity-the total PA score is categorized into levels. This scale has demonstrated content and construct validity in Chinese student populations and has been widely used in epidemiological and educational settings in China (Yang et al., 2026). In the current study, the Cronbach's α coefficient of the scale was 0.73, indicating an acceptable level of internal consistency reliability.

#### 2.3.2 Externalizing problem behaviors measures

The Strengths and Difficulties Questionnaire (SDQ), originally developed by American psychologist Goodman and revised by Kou Jianhua et al. was used to assess externalizing problem behaviors in children (Goodman, 1997). The SDQ is a globally recognized screening tool designed to evaluate adolescents' behaviors and emotions over the past 6 months. Externalizing problem behaviors in children and adolescents are divided into two dimensions: conduct problems and hyperactivity/inattention, with a total of 10 items. Each item is rated on a 0 (not true) to 2 (certainly true) scale, with higher scores indicating a greater level of externalizing problem behaviors. The Chinese version of the SDQ has shown good applicability among adolescents in China (Shan et al., 2025). In this current study, the Cronbach's α coefficient for the scale was 0.83, indicating high internal consistency.

#### 2.3.3 School connectedness

This current study used the School Connectedness Scale developed by (Resnick 1997), which is designed to measure adolescents' level of school connectedness. The scale consists of three dimensions: peer support, teacher support, and school belonging, with a total of 10 items. A 5-point Likert scale was used, ranging from 1 (strongly disagree) to 5 (strongly agree). The total score ranges from 10 to 50, with higher scores indicating a higher level of school connectedness. In this current study, the Cronbach's α coefficient for the scale was 0.91, indicating high internal consistency.

#### 2.3.4 Self-control

The current study used the Self-Control Scale developed by (Tangney et al. 2004) and revised by (Tan and Guo 2008). which includes 19 items measuring impulse control, healthy habits, resistance to temptations, work focus, and restraint from leisure activities. A 5-point Likert scale was used, with responses ranging from 1 (strongly disagree) to 5 (strongly agree), and the total score ranges from 19 to 95, with higher scores indicating stronger self-control ability. In the current study, the Cronbach's α coefficient for the scale was 0.94.

### 2.4 Data analysis methods

The current study utilizes SPSS 27.0 for data processing. The researcher employed Harman's single-factor method to test the common method bias of the data, ensuring the reliability of the data. Descriptive statistics and correlation analysis were then performed. Pearson correlation analysis was used to examine the relationships between variables. A multiple linear regression model was constructed to analyze the main effects, and further testing of the chain mediation effect was conducted using Model 6 in the SPSS macro plugin Process 4.1 developed by (Hayes 2013) (“Mediation, moderation, and conditional process analysis”). The chain mediation effect was tested using the Bootstrap method with a 95% confidence interval and 5,000 samples.

## 3 Results

### 3.1 Common method bias test

First, procedural controls were implemented to avoid common method bias: (1) the scale included appropriately reverse-coded items; (2) the order of the scale items was randomized; (3) participants were informed that the survey would be completed anonymously. Second, Harman's single-factor model was used to assess the extent of common method bias in the data (Podsakoff et al., 2003). The current study indicated that 24 common factors with initial eigenvalues greater than 1 were extracted through principal component analysis, accounting for 61.036% of the total variance. The unrotated cumulative variance percentage for the first factor was 3.716%, which was well below the 40% threshold, suggesting that there was no severe common method bias in the data of this study (Hayes, 2013) (“Mediation, moderation, and conditional process analysis”).

### 3.2 Descriptive statistics and correlation analysis

Table 2 lists and describes the means, standard deviations, and Pearson correlation coefficients for PA, externalizing problems, school connectedness, and self-control. PA levels were categorized as low ( ≤ 19 points), moderate (20–42 points), or high (≥43 points). Among the 517 participants, 61.2% exhibited low physical activity levels, 25.4% had moderate activity, and only 13.5% had high activity levels. This distribution suggested that most adolescents in this sample engage in insufficient physical activity. The mean score for externalizing problems was 10.04 (SD = 1.96). The mean score for school connectedness was 30.19 (SD = 4.12). The mean score for self-control was 55.03 (SD = 4.90).

**Table 2:** Means, standard deviations and correlations among all variables (*N* = 517)^*^.

Variable	M	SD	1	2	3	4
1. Physical activity	14.83	2.94	1	–	–	–
2. Externalizing problems	10.04	1.96	−0.228^**^	1	–	–
3. School connectedness	30.19	4.12	0.184^**^	−0.260^**^	1	–
4. Self-control	55.03	4.90	0.186^**^	−0.221^**^	0.339^**^	1

Pearson correlation coefficients.

^*^p < 0.05; ^**^p < 0.01.

The current study indicated that externalizing problem behaviors were significantly negatively correlated with physical activity (PA) (*r* = −0.228, *p* < 0.01), school connectedness (*r* = −0.260, *p* < 0.01), and self-control (*r* = −0.221, *p* < 0.01). Additionally, PA was positively correlated with both school connectedness (*r* = 0.184, *p* < 0.01) and self-control (*r* = 0.186, *p* < 0.01). Among the mediating variables, school connectedness and self-control were also positively correlated (*r* = 0.339, *p* < 0.01).^*^p < 0.05, ^*^p < 0.01.

### 3.3 Regression analysis

To examine whether PA, school connectedness, and self-control could relate to externalizing problems, hierarchical multiple regression analysis was conducted, with the results presented in Table 3. Prior to conducting regression and mediation analyses, we tested the key statistical assumptions. Scatter plots indicated linear relationships among all variables. Residuals were examined and found to be approximately normally distributed, and no significant outliers were identified in the dataset. Multicollinearity diagnostics showed that all variance inflation factor (VIF) values ranged from 1.31 to 1.60, well below the commonly recommended threshold of 10, indicating no multicollinearity concerns. Overall, all assumptions were adequately satisfied, supporting the validity of the subsequent regression and mediation analyses.

**Table 3:** Results of regression analysis^**^.

Variant		Dependent variable: EP								Dependent variable: SC		Dependent variable: S–C	
		Model 1		Model 2		Model 3		Model 4		Model 5		Model 6	
		β	*t*	β	*t*	β	*t*	β	*t*	β	*t*	β	*t*
Constant		11.37^***^	6.88	13.56^***^	8.14	16.22^***^	9.54	18.12^***^	9.94	24.76^***^	7.02	37.95^***^	9.12
Control Variable	Gender	0.02	0.10	0.07	0.41	0.05	0.30	0.05	0.31	−0.19	−0.53	0.02	0.05
	Age	−0.33	−0.97	−0.29	−0.86	−0.32	−0.97	−0.32	−0.98	−0.25	−0.36	−0.01	−0.01
	Grade level	0.39	1.00	0.32	0.84	0.39	1.04	0.40	1.07	0.61	0.75	0.17	0.19
	Place of birth	−0.18	−0.98	−0.17	−0.94	−0.16	−0.95	−0.12	−0.70	0.03	0.09	0.87^*^	2.06
	Only child or not	0.25	1.36	0.20	1.11	0.18	1.06	0.19	1.08	−0.13	−0.34	0.04	0.09
	Boarding or not	0.03	0.17	0.00	0.01	0.02	0.14	0.03	0.16	0.21	0.58	0.06	0.14
Independent variable	PA	–	–	−0.15^***^	−5.20	−0.12^***^	−4.25	−0.11^***^	−3.86	0.27^***^	4.34	0.22^***^	3.07
Mediating variable	SC	–	–	–	–	−0.11^***^	−5.27	−0.09^***^	−4.17	–	–	0.37^***^	7.41
	S-C	–	–	–	–	–	–	−0.05^***^	−2.77	–	–	–	–
Model fit	*R*	0.09		0.24		0.33		0.35		0.21		0.37	
	*R*2	0.01		0.06		0.11		0.12		0.04		0.14	
	Adjusted*R*2	0.01		0.05		0.09		0.10		0.03		0.12	
	Δ*R*2	0.01		0.06		0.11		0.12		0.04		0.14	
	*F*	0.57		3.19^***^		6.74^***^		6.91^***^		2.82^*^		9.09^***^	

PA, Physical Activity; EP, Externalizing Problems; SC, School Connectedness; S-C, Self-Control.

^*^p < 0.05; ^**^p < 0.01;^***^p < 0.001.

In the regression analysis, demographic variables such as gender, age, grade level, place of birth, whether the participant was an only child, and whether they were boarding or not were included as control variables in Model 1. Next, the independent variable PA was added to Model 2, and the results indicated that it was significantly negatively associated with externalizing problems (β= −0.15, *p* < 0.001). In Model 3, the mediator variable, school connectedness, was further included, and it was also significantly negatively correlated with externalizing problems (β=-0.11, *p* < 0.001). In Model 4, when PA, school connectedness, and the other mediator, self-control, were included, self-control was significantly negatively linked to externalizing problems as well (β = −0.05, *p* < 0.001).

Additionally, in Model 5, where school connectedness was the dependent variable, PA was significantly positively associated with school connectedness (β = 0.27, *p* < 0.01). Finally, the results from Model 6 indicated that PA was significantly positively correlated with self-control (β = 0.22, *p* < 0.01). This hierarchical regression analysis illustrated the interrelationships between PA, school connectedness, self-control, and externalizing problems, with each variable indicating a significant link to externalizing problems.

### 3.4 Mediating role test

**Figure 3:**
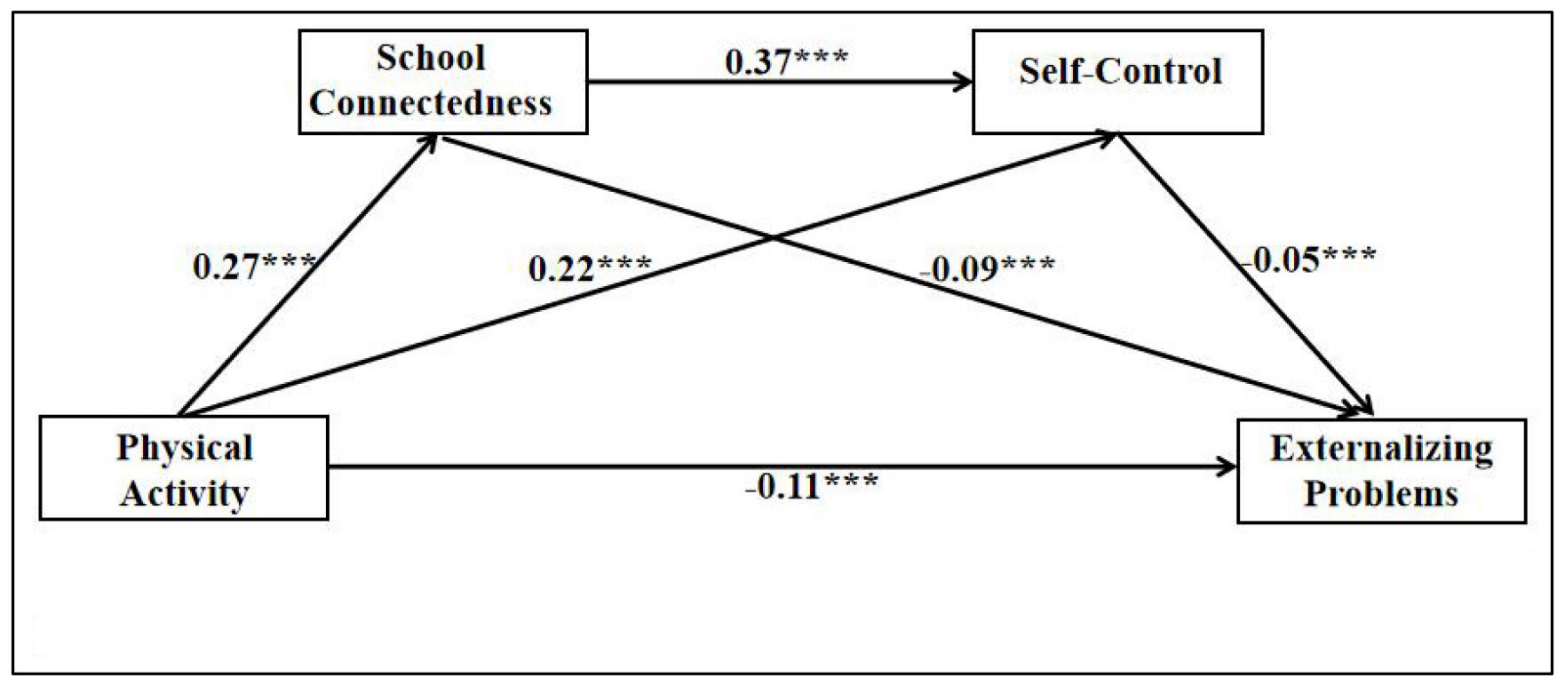
Chain mediation effects of school connectedness and self- control in a relationship between physical activity and externalizing problems. Non-standarad path coefficient. ^***^p < 0.001.

The results of the chained mediation analysis (see Table 4; Figure 3) indicated that PA had a significant direct association with externalizing problems (*b* = −0.11, 95% CI [−0.17, −0.06]), which accounted for 75% of the total effect. Furthermore, there was a significant total indirect association of school connectedness and self-control between PA and externalizing behaviors (*b*=-0.04, 95% CI[−0.06, −0.02]), accounting for 25% of the total effect. These findings suggested the existence of a chain-mediated pathway in which school connectedness and self-control jointly explained the association between PA and externalizing behaviors in adolescents with ADHD, providing empirical support for Hypothesis 4.

**Table 4:** Mediating effects and effect sizes.

Path	Effect	SE	Bootstrap 95%CI		Percentage of total effect
			Lower	Upper	
Total effect	−0.15	0.03	−0.20	−0.11	–
Direct Effect	−0.11	0.03	−0.17	−0.06	75%
Total indirect effects	−0.04	0.01	−0.06	−0.02	25%
Path1:PA → schoolconnectedness → exte-rnalizing problems	−0.02	0.01	−0.04	−0.01	59%
Path2:PA → selfcontrol → externalizing problems	−0.01	0.01	−0.02	−0.01	28%
Path3:PA → school-connectedness → self-control → externalizing problems	−0.01	0.00	−0.02	−0.01	13%

In the path-specific mediation results for Path 1, school connectedness was found to exhibit a significant indirect association in the link between PA and externalizing behaviors (*b* = −0.02, 95% CI[−0.04, −0.01]), accounting for 59% of the total indirect effect. This finding supported Hypothesis 2, indicating that school connectedness functioned as a mediator in the relationship between PA and externalizing behaviors in adolescents with ADHD. Path 2 results indicated that self-control significantly mediated the association between PA and externalizing behaviors (*b* = −0.01, 95% CI[−0.02, −0.01]), accounting for 28% of the total indirect effect. This supported Hypothesis 3, suggesting that self-control played a mediating role in the association between PA and externalizing behaviors in adolescents with ADHD. Path 3 results demonstrated that the sequential mediating role of school connectedness and self-control was significant (*b* = −0.01, 95% CI[−0.02, −0.01]), accounting for 13% of the total indirect effect.

## 4 Discussion

The current study examined the relationships between PA, school connectedness, self-control, and externalizing problems, with a particular focus on the association between PA and externalizing problems in adolescents with ADHD, as well as the sequential mediating roles of school connectedness and self-control in this relationship. The results indicated that PA, school connectedness, and self-control were linked to lower levels of externalizing problems among adolescents with ADHD. Further analysis indicated that school connectedness and self-control jointly formed a chain-mediated pathway, linking PA to externalizing problems in adolescents with ADHD.

### 4.1 The relationship between physical activity and externalizing problems

The results of the current study indicated that PA was significantly negatively associated with externalizing behaviors among adolescents with ADHD. This finding supported the hypothesis that PA may be linked to fewer negative emotions and behavioral difficulties through mechanisms such as neuroplasticity (Stillman et al., 2020). In the adolescent ADHD population, higher levels of PA were significantly negatively correlated with externalizing problems, consistent with Hypothesis 1 (Pan et al., 2016; Conway et al., 2024). This result corroborated previous research, indicating that PA was not only beneficial for adolescents' physical health but also relevant to improved mental health and associated with better emotional wellbeing (Rodriguez-Ayllon et al., 2023). This current study added to the growing body of evidence linking PA to lower levels of externalizing problems in adolescents with ADHD, consistent with previous research. Even after controlling for individual and family-level covariates, the association between PA and fewer externalizing behaviors remained significant, replicating patterns reported in prior studies of adolescents with ADHD (Liang et al., 2024). Overall, these findings highlighted the consistent and meaningful association between PA and lower levels of externalizing symptoms among adolescents with ADHD.

To foster PA participation among adolescents with ADHD, families may consider establishing intergenerational sports transmission models to strengthen parent-child connection. Schools could integrate diverse, structured team sports (e.g., three 45-min basketball or football sessions per week) into the curriculum. Communities could develop trauma-informed sports programs that combine athletic coaching with psychological support, collectively supporting the mental health of adolescents with ADHD.

### 4.2 The mediating role of school connectedness between physical activity and externalizing problems

This current study found that school connectedness partially mediated the relationship between PA and externalizing problems, accounting for 59% of the total effect, supporting Hypothesis 2. This result aligned with findings in typically developing adolescents (TD) (Wilson et al., 2025). In the ADHD adolescent population, high-quality school connectedness was closely associated with fewer externalizing problems, consistent with prior research, which suggested that strong peer relationships and a positive sense of school belonging were significantly linked to fewer problem behaviors and better psychological adaptation (Dvorsky et al., 2018). Specifically, PA was not only directly linked to adolescents' externalizing behaviors but also indirectly associated with them through its correlation with school connectedness (McNamara et al., 2015). First, collective PA may foster emotional bonds and trust among peers through teamwork and shared goals, enhancing peer support. Second, positive peer relationships and a sense of school involvement formed through PA may help adolescents perceive themselves as important members of the school and peer group, thus increasing school belonging (Michael et al., 2024). These associations suggest that school connectedness may play a role in the link between PA and externalizing behaviors in adolescents with ADHD.

Compared to previous studies, our research also found a higher proportion of male adolescents with ADHD in the low school connectedness group, consistent with Lester et al.'s conclusion that male students have weaker school connectedness than females (Lester et al., 2013). Gender differences in peer functioning among adolescents with ADHD may partly stem from higher rates of impulsive, disruptive externalizing behaviors in boys, which render them more vulnerable to peer conflicts; girls with ADHD generally display fewer overt disruptive and impulsive symptoms, resulting in reduced risk of such interpersonal disputes (Dimitri et al., 2025). This current study not only deepens the understanding of the mechanisms behind externalizing behaviors in adolescents with ADHD but also expands the role of school connectedness in special populations, offering a new theoretical perspective for applying social support theory in school settings. In practice, schools could enhance peer acceptance in adolescents with ADHD by optimizing the social design of group sports activities and offering social skills training for male students, thereby supporting greater school connectedness and fewer externalizing problems.

### 4.3 The mediating role of self-control between physical activity and externalizing problems

The current study demonstrated that self-control serves as a significant mediator in the association between PA and externalizing problems in adolescents with ADHD, supporting Hypothesis 3 and aligning with prior research (Cui et al., 2024). Results indicated that PA was positively correlated with self-control, which in turn was negatively associated with externalizing problems. The PA → Self-Control → Externalizing Problems longitudinal mediation model underscores the psychological role of self-control. Consistent with the self-control strength model (Hagger et al., 2010), which posits that self-control is a depletable resource that may be enhanced through practice, PA may be linked to better self-regulation and fewer externalizing problems in ADHD adolescents (Finne et al., 2019; Piquero et al., 2010).

Unlike prior studies focusing on the correlates of PA and self-control with adolescent behavior, our research highlights the mediating role of self-control between PA and externalizing behaviors. Additionally, the present study demonstrated that individuals with low trait self-control are more likely to engage in aggressive behaviors when fatigued, especially in the ADHD adolescent group (DeWall et al., 2007). In practice, schools could design engaging and structured physical activities, such as team sports and mindfulness-based training, which may support self-control and reduce the likelihood of externalizing behaviors in adolescents with ADHD.

### 4.4 Chain mediation of school connectedness and self-control between physical activity and externalizing problems

This current study indicated that school connectedness and self-control were jointly associated with the link between PA and externalizing behaviors, supporting Hypothesis 4. Results indicated that school connectedness was significantly positively associated with self-control in adolescents with ADHD, which in turn was related to fewer externalizing problems. This current study was consistent with prior research (Hawkins and Weis, 2017). Specifically, school connectedness was linked to greater school belonging and academic identification in adolescents with ADHD, which may correlate with better emotional regulation and behavioral management, and thus with better self-control. The current study aligned with the protection-enhancement hypothesis, suggesting that structured PA associated with a high sense of belonging was linked to better teacher-student relationships, peer support, and emotional support, which correlated with greater school engagement and participation willingness, in turn associated with better self-control and fewer aggressive behaviors (Wills et al., 2002).

School connectedness, through its correlation with self-control, was associated with fewer externalizing problems in adolescents with ADHD. This current study enriched the body-environment interaction framework and provided a fresh perspective on the behavioral development of adolescents with ADHD. In practice, schools could improve school connectedness by optimizing teaching methods and strengthening teacher-student interactions. Additionally, team sports like basketball and football may further support self-control. For those with weak self-control, schools could provide behavioral counseling and psychological support, using both environmental and individual approaches to help reduce the likelihood of externalizing problems and support positive development in adolescents with ADHD.

### 4.5 Research limitations and future perspectives

This current study has several limitations related to its measurement approach. Firstly, the current study did not differentiate among types, intensities, or contexts of physical activity. The measure only captured overall activity level, leaving it unclear whether specific forms of activity are differently associated with externalizing behaviors. Future research could build on these findings by examining the unique contributions of different activity types.

Secondly, the sample was recruited from a limited number of local schools and rehabilitation centers, and adolescents with ADHD may vary by region or type of educational setting. Therefore, the results may not fully represent the general population of adolescents with ADHD. Future studies with more diverse and geographically broader samples are needed to confirm and extend these findings.

Thirdly, the cross-sectional design limits causal inferences about the sequential relationships among physical activity, school connectedness, self-control, and externalizing problems. Although the proposed model is theoretically sound, future research should employ longitudinal, experimental, or diary-based designs to examine the temporal and causal dynamics of these pathways.

Finally, all constructs were assessed using self-report measures. Although procedural and statistical controls were applied, this may increase the risk of common method bias. Future research could benefit from using multiple methods, such as objective physical activity indicators (e.g., accelerometers or wearable devices), multi-informant reports (e.g., teachers or parents), or behavioral tasks, to enhance the robustness of the measurements.

## 5 Conclusion

This current study incorporated the variables of school connectedness and self-control to explore the different pathways through which PA was associated with externalizing problems in adolescents with ADHD from both the school and individual perspectives. The results indicated that both factors were linked to fewer externalizing problems in adolescents with ADHD, offering new theoretical insights into the relationship between PA and externalizing problems in youth. Furthermore, school connectedness and self-control were associated with the link between PA and externalizing problems in adolescents with ADHD, indicating a sequential pattern of associations. This current study highlights the significant value of these factors in adolescent psychopathology.

## Data Availability

The original contributions presented in the study are included in the article/supplementary material, further inquiries can be directed to the corresponding author.
